# Relative efficacy of egg yolk and soya milk-based extenders for cryopreservation (−196°C) of buffalo semen

**DOI:** 10.14202/vetworld.2015.239-244

**Published:** 2015-02-25

**Authors:** D.V. Chaudhari, A. J. Dhami, K. K. Hadiya, J. A. Patel

**Affiliations:** 1 Sabarmati Ashram Gaushala, Ahmedabad, Gujarat, India; 2 Department of Animal Reproduction, Gynaecology and Obstetrics, College of Veterinary Science and Animal Husbandry, Anand Agricultural University, Anand, Gujarat, India

**Keywords:** buffalo semen, cryopreservation, extenders, egg yolk, relative efficacy, soybean milk

## Abstract

**Aim:**

The aim was to compare commercially available soybean milk-based extenders, *viz*. Bioxcell^®^ and Optixcell^®^ (IMV, France) with standard Tris-citrate-fructose-egg yolk-glycerol (TFYG) extender for cryopreservation of buffalo semen.

**Materials and Methods:**

Semen was collected twice a week in artificial vagina from six sexually mature, 4-6 years old, healthy breeding bulls of Surti buffalo breed. In all 48 qualifying ejaculates (8 per bull) having initial motility >70% were split into three equal aliquots and were diluted (at 34°C keeping 100×10^6^ sperm ml^−1^) in TFYG, Bioxcell and Optixcell extenders. The French mini straws filled from each aliquot were gradually cooled to 4-5°C, equilibrated at 4°C for 4 h and frozen in liquid nitrogen 2 vapor using programmable biofreezer. Just before freezing (post-equilibration) and 24 h after frozen storage, the samples were evaluated for various sperm quality parameters using standard protocols. Frozen semen straws were thawed in a water bath at 37°C for 30 s. The post-thaw incubation survival (37°C for 1 h) was assessed through motility rating at 0, 30 and 60 min of incubation.

**Results:**

The mean percentages of prefreeze sperms in TFYG, Bioxcell and Optixcell extenders in terms of progressive motility (69.48±0.37, 68.02±0.49, 70.94±0.38), viability (79.21±0.39, 77.38±0.48, 81.58±0.38), total abnormalities (7.90±0.14, 8.60±0.16, 7.08±0.15), intact acrosome (89.54± 0.18, 88.58±0.22, 90.52±0.21) and hypoosmotic swelling (HOS) reactivity (67.96±0.32, 65.65±0.42, 70.23±0.37) varied significantly (p<0.05) between extenders. Similar pattern of significant (p<0.05) variations between these extenders for post-thaw sperm progressive motility (47.71±0.79, 44.38±0.85, 49.90±0.90), viability (57.19±0.79, 53.85±0.84, 59.67±0.91), total abnormalities (12.33±0.17, 12.75±0.21, 11.27±0.18), intact acrosome (76.83±0.23, 75.90± 0.27, 78.50±0.25) and HOS reactivity (45.02±0.84, 42.31±0.82, 47.81±0.90) was also observed for TFYG, Bioxcell and Optixcell extenders. The recently launched improved soybean milk-based extender Optixcell excelled the older Bioxcell extender and even standard TFYG in respect of some of the sperm quality parameters.

**Conclusion:**

The advantages of soy lecithin-based bovine semen extenders over egg yolk regarding sanitary issues are unquestionable but still egg yolk-based semen extenders are widely used because of the cost factor and good *in vivo* fertility results.

## Introduction

Semen cryopreservation and artificial insemination (AI) offer many advantages to the livestock industry, particularly in conjunction with genetic evaluation and selection programs [1]. Worldwide application of AI was the main impulse for subsequent expansion of other procedures, e.g. management of estrus, freezing of sperm, sperm sexing, embryo manipulation and cloning [2]. Egg yolk has been used as a basic component of extenders for bovine ejaculate since 1939 [3] and has still remained popular. Use of these extenders is recommended because of their excellent protection of sperm cells [4]. Nevertheless, the use of egg yolk as a cryoprotectant has recently been restricted in some countries for reasons of immunologic and hygiene risks [5]. Moreover, some researchers [6] have demonstrated that extenders based on egg yolk can have negative effects on sperm respiration and motility due to other specific substances they contain. There is an increased global concern regarding microbiological safety. Therefore, recent studies are in progress aiming to develop chemically defined extenders, free of compounds of animal origin. Despite the significant benefits of egg yolk and milk on semen cryopreservation, such components of animal origin may represent a potential microbiological risk, compromising the quality of cryopreserved semen and standardization.

An alternative to replace the components of animal origin in semen extenders is the soy lecithin, a natural mixture of phosphatidyl choline and several fatty acids such as stearic, oleic, and palmitic. Such fatty acids, the prevailing phospholipids in most of the mammalian biological membranes, are known to confer structural stability to cells [7]. Due to such composition, studies aiming to evaluate the efficiency of soy lecithin as a primary source of lipoproteins in semen extenders were performed in bovine [8-10], buffalo (bubaline) [11], ovine [12] and equine [13]. However, results obtained using lecithin as substitutes to egg yolk are still a matter of debate [14]. Furthermore, due to the reduced technological innovations on semen cryopreservation over the last years [4], the Tris-egg yolk-fructose extender is still the most commonly employed worldwide.

The present study was therefore aimed to evaluate the comparative efficacy of routine egg yolk and commercial soybean-based extenders for cryopreservation of buffalo semen based on pre-freeze and post-thaw sperm quality parameters.

## Materials and Methods

### Ethical approval

No ethical approval was necessary to pursue this research work. However the semen collection was done as per standard procedure without harming the animals.

### Semen collection and evaluation

The study was conducted during favorable breeding season on semen of six sexually mature healthy Surti buffalo bulls (*Bubalus bubalis*), 4-6 years old. The bulls were maintained under uniform standard nutritional and managerial practices at the Central Sperm Station of the College in Anand, Gujarat (India). The bulls were under regular twice a week semen collection schedule using an artificial vagina. Semen collections were made in the early morning between 7.30 h and 8.30 h. Two consecutive ejaculates were obtained using an artificial vagina at each collection attempt with 10-12 min interval and pooled samples were used for further study. The ejaculates (8 per bull, total 48) after collection were immediately transferred in to a water bath at 34°C and evaluated for gross quality, motility, and sperm concentration (by Accucell photometer). Only the pooled ejaculates with >70% initial motility were used for further processing and freezing.

### Preparation of extenders

The standard Tris-citrate-fructose-egg yolk-glycerol (TFYG) extender containing 73 ml of Tris buffer (Tris 3.048 g, citric acid 1.67 g, fructose 1.25 g in 100 ml Milli-Q water) + 20 ml fresh egg yolk + 7 ml glycerol was prepared fresh daily as per FAO (1979) and antibiotics benzyl penicillin 1000 IU/ml and streptomycin sulfate 1000 µg/ml were added as recommended. The commercial Bioxcell^®^ and Optixcell^®^ were prepared fresh for use by diluting 2 and 4 times, respectively, with Milli-Q water according to manufacturer’s instructions (IMV, France). The commercial extenders contain carbohydrates, mineral salts, buffer, antioxidant, phospholipids, ultra-pure water, glycerol and antibiotics (gentamicin, tylosin, lincomycin, and spectinomycin) conforming to European Regulations CEE 88/407. They do not contain protein of animal origin but have plant proteins.

### Semen processing

Qualifying pooled ejaculates from each bull were split into three equal aliquots and diluted in single step at 34°C with each of three experimental extenders @ 100 × 10^6^ spermatozoa ml^−1^ and were evaluated for progressive sperm motility. Then the French mini straws (at least 10) were filled from each aliquot using automatic filling and sealing machine (IS4 System, IMV Technologies, France) and cooled to 4-5°C within 60-90 min and further equilibrated at the same temperature for 4 h in cold handling cabinet. After equilibration, the samples were evaluated for various sperm quality parameters, *viz*., motility, viability, morphology, acrosome integrity and HOS test using standard procedures and phase contrast microscope. Freezing of straws was carried out in liquid nitrogen (LN_2_) vapor using a programmable bio-freezer (Digitcool 5300 CE ZH 350, IMV Technologies, France). The straws were then plunged in LN_2_ (−196°C) for overnight storage. Semen straws were thawed next day in a water bath at 37°C for 30 s.

### Assessment of sperm quality

The sperm progressive motility was determined at 37°C temperature under high power magnification (×40) of phase contrast microscope (Longshou, USA) fitted with a biotherm stage and a closed circuit television. The viability of spermatozoa was assessed with eosin-nigrosin stained semen smears [15] under oil emulsion lens of a phase contrast microscope (×100; Olympus BX20, Tokyo, Japan). Simultaneously, sperms were also examined for total and various types of segmental abnormalities of the head, mid-piece, and tail region. The percentages of spermatozoa with intact acrosome and those with different forms of acrosomal abnormalities were assessed using Geimsa stain according to Watson [16]. The plasma membrane integrity of spermatozoa was assessed using a HOS test employing 150 mOs/L solution of sodium citrate and fructose with 30 min of incubation at 37°C [17,18]. At the end of incubation, 5 μl of formalized eosin solution (10%) was added in order to stop the reaction, and stain and fix the sperm cells. The wet preparations of semen were then evaluated using a phase contrast microscope (×40). Nearly 200 spermatozoa were assessed from different fields for each trait and the spermatozoa with various forms of morphological defects, acrosome defects, and swollen coiled tail were counted and expressed as percentage.

### Statistical analysis

The data generated were analyzed statistically using ANOVA and critical different test by employing IBM SPSS Statistics version 17.00 to know the effect of extenders, as well as processing steps on various sperm quality traits evaluated.

## Results and Discussion

We evaluated the comparative efficacy of conventional standard egg-yolk based extender (TFYG) and commercially available soybean-based extenders (Bioxcell and Optixcell, IMV, France) for cryopreservation of Surti buffalo bull semen in terms of percentage motility, viability, morphology, acrosome integrity and HOS reactivity at initial, pre-freeze (post-equilibration) and post-freezing. The results are presented in Tables-1-3. The statistical analysis of data revealed that there were significant differences in the levels of these parameters between semen extenders (p<0.05) and between stages (p<0.01) of freezing.

**Table-1:** Mean (±SE) values (%) of sperm parameters in Surti buffalo bulls’ semen at different stages of cryopreservation (−196°C) in different extenders

Freezing stage	Extender	Progressive motility %	Live sperm %	Morphological normal sperm %	Intact acrosome %	HOS reactive sperm %
Initial	-	78.54^a^±0.30	90.48^a^±0.19	93.85^a^±0.08	94.40^a^±0.12	79.35^a^±0.24
Prefreeze	TFYG	69.48^q^±0.37	79.21^q^±0.39	92.10^q^±0.14	89.54^q^±0.18	67.96^q^±0.32
	Bioxcell	68.02^r^±0.49	77.38^r^±0.48	91.40^r^±0.16	88.58^r^±0.22	65.65^r^±0.42
	Optixcell	70.94^p^±0.38	81.58^p^±0.38	92.92^p^±0.15	90.52^p^±0.21	70.23^p^±0.37
	Average	69.48^b^±0.26	79.39^b^±0.28	92.14^b^±0.10	89.55^b^±0.14	67.94^b^±0.26
Postthaw	TFYG	47.71^x^±0.79	57.19^y^±0.79	87.67^y^±0.17	76.83^y^±0.23	45.02^y^±0.84
	Bioxcell	44.38^y^±0.85	53.85^z^±0.84	87.25^y^±0.21	75.90^z^±0.27	42.31^z^±0.82
	Optixcell	49.90^x^±0.90	59.67^x^±0.91	88.73^x^±0.18	78.50^x^±0.25	47.81^x^±0.90
	Average	47.33^c^±0.52	56.90^c^±0.52	87.88^c^±0.12	77.08^c^±0.17	45.05^c^±0.52

Means bearing different superscripts between freezing stages (a,b,c) and between extenders at prefreeze (p, q, r) and postthaw (x, y, z) stage differ significantly (p<0.05), TFYG=Triscitratefructoseegg yolkglycerol, SE=Standard error

**Table-2:** Mean (±SE) values (%) of segment wise sperm abnormalities in Surti buffalo bulls’ semen at different stages of cryopreservation (−196°C) in different extenders

Freezing stage	Extender	Sperm segments			Overall

		Head	Mid-piece	Tail	
Initial	-	1.83^a^±0.05	0.88^a^±0.03	3.44^a^±0.05	6.15^a^±0.08
Prefreeze	TFYG	2.27^p^±0.08	1.23^q^±0.06	4.40^q^±0.07	7.90^q^±0.14
	Bioxcell	2.19^p^±0.09	1.56^p^±0.07	4.85^p^±0.12	8.60^p^±0.16
	Optixcell	1.79^q^±0.08	1.08^q^±0.08	4.21^q^±0.11	7.08^r^±0.15
	Average	2.08^b^±0.05	1.29^b^±0.04	4.49^b^±0.06	7.86^b^±0.10
Postthaw	TFYG	3.42^x^±0.09	2.08^x^±0.07	6.83^x^±0.10	12.33^x^±0.17
	Bioxcell	3.48^x^±0.10	2.23^x^±0.09	7.06^x^±0.12	12.75^x^±0.21
	Optixcell	3.02^y^±0.11	1.79^y^±0.07	6.46^y^±0.12	11.27^y^±0.18
	Average	3.31^c^±0.06	2.03^c^±0.05	6.78^c^±0.07	12.12^c^±0.12

Means bearing different superscripts between freezing stages (a,b,c) and between extenders at prefreeze (p,q,r) and postthaw (x,y,z) stage differ significantly (p<0.05) TFYG=Triscitratefructoseegg yolkglycerol, SE=Standard error

**Table-3:** Mean (±SE) values (%) of various types of acrosomal abnormalities in Surti buffalo bulls’ semen at different stages of cryopreservation (−196°C) in different extenders

Freezing stage	Extender	Sperm acrosomal abnormalities				Overall

		Swollen	Ruffled	Detached	Denuded	
Initial	-	2.29^a^±0.05	1.60^a^±0.06	1.10^a^±0.05	0.60^a^±0.05	5.60^a^±0.12
Prefreeze	TFYG	3.79^q^±0.11	2.90^p^±0.09	2.27±0.09	1.50^p^±0.09	10.46^q^±0.18
	Bioxcell	4.21^p^±0.10	2.96^p^±0.08	2.52±0.08	1.69^p^±0.07	11.42^p^±0.22
	Optixcell	3.46^r^±0.12	2.56^q^±0.10	2.31±0.09	1.17^q^±0.05	9.48^r^±0.21
	Average	3.82^b^±0.07	2.81^b^±0.05	2.37^b^±0.05	1.45^b^±0.05	10.45^b^±0.14
Postthaw	TFYG	8.50^x^±0.15	6.17^y^±0.11	5.13±0.13	3.31^x^±0.11	23.17^y^±0.23
	Bioxcell	8.65^x^±0.15	6.71^x^±0.13	5.25±0.11	3.50^x^±0.11	24.10^x^±0.27
	Optixcell	7.81^y^±0.18	5.83^z^±0.11	5.00±0.12	2.81^y^±0.10	21.50^z^±0.25
	Average	8.32^c^±0.10	6.24^c^±0.07	5.13^c^±0.07	3.21^c^±0.07	22.92^c^±0.17

Means bearing different superscripts between freezing stages (a,b,c) and between extenders at prefreeze (p,q,r) and postthaw (x,y,z) stage differ significantly (p<0.05), TFYG=Triscitratefructoseegg yolkglycerol, SE=Standard error

The overall mean percentages of progressively motile spermatozoa observed initially in fresh buffalo semen declined progressively and statistically (p<0.01) after equilibration (pre-freeze stage) and freezing (post-thaw stage). The values observed at pre-freeze and post-thaw stages in Optixcell extender were significantly higher than in Bioxcell extender, and the values in TFYG extender were intermediate (Table-1). The overall mean percentages of motile spermatozoa immediately after thawing (0-min), and after 30- and 60-min of post-thaw incubation at 37°C were 47.33±0.85, 41.67±0.85 and 34.62±0.82 (p<0.01), respectively. The overall post-thaw incubation survival was significantly (p<0.05) better in Optixcell, followed by TFYG as compared to Bioxcell extender at all the stages of evaluation. The results showed that the commercial soya-based Optixcell as well as standard egg yolk-based TFYG extenders could sustain better and acceptable level of sperm survival at least for 1 h after thawing (37.19±0.81 and 35.52±0.79% vs. 31.15±0.85%), hence semen frozen in such extenders can be used successfully in AI with better anticipated conception rates.

The overall mean percentages of live and morphologically normal spermatozoa also followed the pattern of sperm motility at initial, pre-freeze and post-thaw stage of buffalo semen, being reduced significantly (p<0.01) at each stage of freezing process. At both pre-freeze and post-thaw stage, Optixcell could maintain greater viability and morphology of sperms as compared to Bioxcell, and TFYG behaved intermediately (Table-1).

The overall mean percentages of sperms with total, head, midpiece and tail abnormalities recorded initially were 6.15±0.08, 1.83±0.05, 0.88±0.03 and 3.44±0.05, respectively, which were increased significantly after equilibration and after freezing-thawing of extended semen. Significantly (p<0.05) lower sperm abnormalities were noted in Optixcell as compared to Bioxcell and even TFYG extender at both pre-freeze as well as post-thaw stages. The same trend was observed in segment-wise sperm abnormalities of the head, midpiece and tail region also (Table-2).

The overall mean percentages of spermatozoa with intact acrosomes and HOS reactivity also declined significantly (p<0.01) at pre-freeze (after equilibration) and post-thaw (after freezing) stage of semen. The acrosome integrity and HOS reactive sperm were maintained at significantly higher level in Optixcell extender as compared with other two extenders at both pre-freeze and post-thaw stages (Table-1). Further, the overall mean percentages of sperms with swollen, ruffled, detached and denuded acrosome, and total damaged acrosomes recorded initially were 2.29±0.05, 1.60±0.06, 1.10±0.05, 0.60±0.05 and 5.60±0.12, respectively, which increased significantly after equilibration and freezing-thawing of extended buffalo semen. The values differed significantly (p<0.01) between stages/periods of cryopreservation and between extenders at each stage, the values being lowest (p<0.05) in Optixcell extender and highest in Bioxcell. The same trend was also observed in the percentage incidence of a particular form of acrosomal abnormality (Table-3).

The present findings of sperm quality parameters observed in buffalo semen at different stages of cryopreservation with egg yolk and soybean-based semen extenders coincided with many previous reports [2,5,8,11,14,19,20]. The findings regarding the performance of TFYG extender and Bioxcell extender were supported by Beran *et al*. [2], who found better post-thaw motility, viability and post-thaw longevity in semen extended with egg yolk-based extender than Bioxcell extender. Our results are also comparable with the earlier report [11], wherein non-significantly higher values for post-thaw motility, viability and less sperm abnormalities were found for semen frozen in TFYG than Bioxcell extender, while our findings showed significantly better quality parameters in semen frozen in TFYG than Bioxcell extender. Further, they also found similar *in vivo* fertility results for both the extenders.

Present findings regarding greater effectiveness of Tris-egg yolk-based extender than Bioxcell extender in preserving progressive motility, and acrosomal as well as plasma membrane integrity are in accordance with the previous findings [14]. Celeghini *et al*. [4] reported that egg yolk-based extender was more effective at preserving total and progressive sperm motility than Bioxcell, which is in agreement with our findings. Apparent differences between the results of Celeghini *et al*. [4] and Leite *et al*. [14] have been due to differences in cooling and freezing rates, and type of extender employed, but is also likely due to differences in breeds of bull. There are breed differences in semen freezability; in particular, European breeds have superior freezability than Zebu breeds [21], probably due to a longer selection time for reproductive traits.

In earlier studies, sperm cryopreserved in soybean-based extenders (Bioxcell, Biociphos-Plus, Botu-Bov-Soy etc.) showed higher straightness and linearity when compared to TFYG extender, a decrease on post-thaw sperm integrity was observed in Bioxcell when compared to TFYG extender [19,22,23]. These varying findings could have been due to differences in extender density, viscosity or even the presence of large particles in different extenders [4]. Munoz *et al*. [10] suggested that Bioxcell extender has less protective effect on maintaining spermatozoal plasma membrane integrity, and indirectly reflected on sperm motility, which was in agreement with our findings. Akhter *et al*. [11] also found non-significantly higher values for plasma membrane integrity and acrosomal integrity of spermatozoa after thawing in semen frozen in TFYG than Bioxcell extender, while our findings showed significantly better quality parameters in semen frozen in TFYG than Bioxcell extender. The results of the present study on plasma membrane and acrosomal integrity, however, are contrary to the earlier report [24], wherein higher post-thaw motility, viability, acrosomal integrity and plasma membrane integrity of sperm were recorded immediately after thawing as well as after 6 h of incubation in Bioxcell extender than in standard TFYG extender.

The advantages of lecithin-based bovine semen extenders over milk and/or egg yolk regarding sanitary issues are unquestionable. According to Bousseau *et al*. [9], the use of lecithin may prevent the contamination with bacteria and mycoplasma. The efficacy of lecithin-based extenders is still a matter of debate. Previous studies report similar or higher sperm motility and plasma membrane and acrosomal integrity [6,8] with similar or even higher fertility rates [9,11,22] for semen cryopreserved using lecithin-based commercial extenders, developed for use with plant-derived compounds. On the other hand, studies of many authors proved better efficiency of egg yolk-based extenders over soya lecithin-based extenders [4,19,25,26].

While comparing TFYG and soya lecithin-based AndroMed extenders, Aires *et al*. [8] favored soya lecithin extenders in terms of good quality parameters and higher conception rate. Similarly Meena *et al*. [20] also supported soybean-based Biociphos extender than TFYG because of better visualization and low bacterial load. However, contradictory findings of Thun *et al*. [5] regarding comparison of TFYG with soybean-based Biociphos-plus revealed better protective capacity of egg yolk-based TFYG extender and higher *in vivo* fertility results as well. Veerabramhaiah *et al*. [26] also supported TFYG extender than Biociphos-plus extender; similarly Crespilho *et al*. [19] proved better cryoprotectivity of egg yolk-based extenders over soya lecithin-based Botu-Bov-Soy lecithin extender.

The considerably reduced values for sperm motility, viability, morphology, and plasma membrane/acrosome integrity observed after cryopreservation of semen over fresh or pre-freeze stage in the present study have also been reported by other researchers. The level of this reduction differed depending on the nature of extender employed, which might indicate that compounds present in the medium had different effects on sperm structures and protective capacity. The results of extender effects of the present study confirmed the observations of many authors, who reported the effects that extender compounds have on sperm motility, membrane integrity and various sperm abnormalities in bovine spermatozoa cooled to 5°C and then frozen.

In our findings newly launched ready to use commercial soybean-based extender ‘Optixcell’ proved better than TFYG and Bioxcell in terms of visualization and quality parameters to some extent as well. Bioxcell extender though soya protein-based had less protective capacity. According to Singh *et al.*, [27] and Rehman *et al*. [28] optimal soya lecithin concentration in the extender is prerequisite for protection of spermatozoa during temperature variations. Concentration of soybean below or above the optimal may be harmful, and this might be the situation with Bioxcell extender. Optixcell is newly launched product and proved better than Bioxcell in our experiment. Ultimately, the composition of semen extenders strongly influences sperm survival [25]. The exact composition of any commercial extender is not disclosed by the manufacturer; otherwise it would be easier to draw a valid conclusion based on the findings.

In the present study, Optixcell gave relatively better performance than the TFYG, but the differences at most instances were not significant, thus we cannot underestimate TFYG extender, as it gives good or satisfactory results as reported by most workers in India and abroad. The only problem with TFYG is regarding the need to prepare fresh daily and the presence of egg yolk, which is likely to introduce the risk of the xenobiotic contamination in the extended semen.

## Conclusions

Significantly (p<0.05) higher sperm progressive motility, viability, acrosomal integrity and plasma membrane integrity with fewer sperm abnormalities were observed at all stages of cryopreservation of buffalo semen extended with Optixcell extender than Bioxcell extender and the TFYG was the intermediate of the two commercial extenders. Problems associated in using traditional egg yolk-based extenders, including bacterial or xenobiotic contamination and variability in composition of egg yolk can be avoided using chemically defined animal protein-free extender Optixcell^®^ (IMV, France) for successful cryopreservation of buffalo semen. The advantages of soy lecithin-based bovine semen extenders over egg yolk regarding sanitary issues are unquestionable but still egg yolk-based semen extenders are widely used by most of AI centers because of cost factor and good *in vivo* fertility results. The recently launched improvised soy milk-based extender Optixcell excelled the older Bioxcell extender in respect of all the sperm quality parameters evaluated through cryopreservation of split-ejaculates. However, further studies are warranted to standardize the chemically defined soybean-based commercial extenders for buffalo semen preservation as a replacement of animal protein source with plant protein source through extensive *in vitro* studies, including *in vivo* fertility trials.

## Authors’ Contributions

AJD planned and designed the study. The experiment was conducted, and laboratory work was done by DVC, KKH, and JAP. All authors participated in data analysis, preparation of draft and revision of the manuscript. All authors read and approved the final manuscript.

## References

[1] Mahmoud K.G.M, Sokary A.A.E, Roos M.E.A, Ghaffar A.D.A, Nawito M (2013). Sperm characteristics in cryopreserved buffalo bull semen and field fertility. Iran. J. Appl. Anim. Sci.

[2] Beran J, Stadnik L, Bezdicek J, Louda F, Citek J, Duchacek J (2012). Effect of sire and extender on sperm motility and share of live or dead sperm in bulls’ fresh ejaculate and in AI doses after thawing. Arch. Tierz.

[3] Amirat L, Tainturier D, Jeanneau L, Thorin C, Gerard O, Courtens J, Anton M (2004). Bull semen *in vitro* fertility after cryopreservation using egg yolk LDL:A comparison with Optidyl a commercial egg yolk extender. Theriogenology.

[4] Celeghini E.C.C, Arruda R.P, Andrade A.F.C, Nascimento J, Raphael C.F, Rodrigues P.H.M (2008). Effects that bovine sperm cryopreservation using two different extenders has on sperm membranes and chromatin. Anim. Reprod. Sci.

[5] Thun R, Hurtado M, Janett F (2002). Comparison of biociphos-plus and tris egg yolk extender for cryopreservation of bull semen. Theriogenology.

[6] Amirat L, Anton M, Tainturier D, Chatagnon G, Battut I, Courtens J (2005). Modifications of bull spermatozoa induced by three extenders:Biociphos, low density lipoprotein and Triladyl, before, during and after freezing and thawing. Reproduction.

[7] Oke M, Jacob J.K, Paliyath G (2010). Effect of soy lecithin in enhancing fruit juice/sauce quality. Food Res. Int.

[8] Aires V.A, Hinsch K.D, Schloesser F.M, Bogner K, Schloesser S.M, Hinsch E (2003). *In vitro* and *in vivo* comparison of egg yolk-based and soybean lecithin based extenders for cryopreservation of bovine semen. Theriogenology.

[9] Bousseau S, Billard J.P, Marquan-Le Guienne B, Gurien B, Camus A, Lechat M (1998). Comparison of bacteriological qualities of various egg yolk sources and the *in vitro* and *in vivo* fertilizing potential of bovine semen frozen in egg yolk free or lecithin based diluents. Theriogenology.

[10] Munoz O, Briand L, Diaz T, Vasquez L, Schmidt E, Desherces S, Anton M, Bencharif D, Tainturier D (2009). Effect of semen dilution to low-sperm number per dose on motility and functionality of cryopreserved bovine spermatozoa using low-density lipoproteins (LDL) extender:comparison to Triladyl and Bioxcell. Theriogenology.

[11] Akhter S, Ansari M.S, Rakha B.A, Andrabi S.M.H, Iqbal S, Ullah N (2010). Cryopreservation of buffalo (Bubalus bubalis) semen in Bioxcell®extender. Theriogenology.

[12] Gil J, Lundeheim N, Soderquist L, Rodriguez-Martinez H (2003). Influence of extender, temperature, and addition of glycerol on post-thaw sperm parameters in ram semen. Theriogenology.

[13] Papa F.O, Felıcio G.B, Melo C.M, Vita B, Avanzi B.R, Aqua J.A (2010). Effect of substituting soybean lecithin for egg yolk in an extender used for the cryopreservation of stallion semen. Anim. Reprod. Sci.

[14] Leite T.G, Filhoa V.R, Arrudab R.P, Andradeb A.F, Emericka L.L, Zaffalonb F.G, Martinsa J.A, Andradec V.J (2010). Effects of extender and equilibration time on post-thaw motility and membrane integrity of cryopreserved Gyr bull semen evaluated by CASA and flow cytometry. Anim. Reprod. Sci.

[15] Campbell R.G, Hancock J.L, Rothscild L (1953). Counting live and dead spermatozoa. J. Exp. Biol.

[16] Watson P.F (1975). Use of Giemsa stain to detect changes in the acrosome of frozen ram spermatozoa. Vet. Rec.

[17] Jayendran R.S, Van der Ven H.H, Perez-Pelaez M, Crabo B.G, Zaneveld L.J.D (1984). Development of an assay to assess the functional integrity of the human sperm membrane & its relationship to other semen characteristics. J. Reprod. Fertil.

[18] Rasul Z, Anzar M, Jalali S, Ahmad N (2000). Effect of buffering system on post-thaw motion characteristics, plasma membrane integrity and acrosome morphology of buffalo spermatozoa. Anim. Reprod. Sci.

[19] Crespilho A.M, Filho M.F, Aqua J.A, Nichi M, Monteiro G.C, Avanzi B.R, Martins A, Papa F.O (2012). Comparison of *in vitro* and *in vivo* fertilizing potential of bovine semen frozen in egg yolk or new lecithin based extenders. Livest. Sci.

[20] Meena G.S, Raina V.S, Gupta A.K, Mohanty T.K, Bishist R (2010). Comparative performance of biociphos and tris-egg yolk based extenders for buffalo semen preservation. Indian J. Anim. Sci.

[21] Anchieta M.C, Filho V.R, Colosimo E, Sampaio I.B.M, Andrade V.J (2005). Semen discarded and freezing ability of Zebu and European bulls from a Brazilian artificial insemination center. Arq. Bras. Med. Vet. Zootec.

[22] Gil J, Januskauskas A, Haard M, Johanisson A, Soderquist L, Rodriguez-Martinez H (2000). Functional sperm parameters and fertility of bull semen extended in Biociphos-Plus and Triladyl. Reprod. Domest. Anim.

[23] Vera O, Amirat L, Diaz T, Vasquez L, Schmidt E, Desherces S, Anton M, Bencharif D, Tainturier D (2009). Effect of semen dilution to low-sperm number per dose on motility and functionality of cryopreserved bovine spermatozoa using low-density lipoproteins (LDL) extender:Comparison to triladyl and bioxcell. Theriogenology.

[24] Asr S.J, Beheshti R, Kohram H (2011). The evaluations of Tris-citrate-egg yolk or Bioxcell extenders on the post-thawed buffalo sperm parameters. Ann. Biol. Res.

[25] Chaveiro A, Machado L, Frijters A, Engel B, Woelders H (2006). Improvement of parameters of freezing protocol for bull sperm using two osmotic supports. Theriogenology.

[26] Veerabramhaiah K, Rao A.S, Rao V.H, Naidu K.V, Rao S.T.V (2011). Efficacy of the tris and biociphos-plus extenders on the freezability of punganur bull semen. Indian J. Anim. Reprod.

[27] Singh V.K, Singh A.K, Kumar R, Atreja S.K (2013). Development of soya milk extender for semen cryopreservation of Karan Fries (crossbreed cattle). Cryo Letters.

[28] Rehman F.U, Qureshi M.S, Khan R.U (2014). Effect of soybean based extenders on sperm parameters of Holstein-Friesian bull during liquid storage at 4ºC. Pak. J. Zool.

